# Challenges in predicting stabilizing variations: An exploration

**DOI:** 10.3389/fmolb.2022.1075570

**Published:** 2023-01-05

**Authors:** Silvia Benevenuta, Giovanni Birolo, Tiziana Sanavia, Emidio Capriotti, Piero Fariselli

**Affiliations:** ^1^ Department of Medical Sciences, University of Torino, Torino, Italy; ^2^ Department of Pharmacy and Biotechnology (FaBiT), University of Bologna, Bologna, Italy

**Keywords:** protein stability, single-point mutation, stability predictors, machine learning, stabilizing variants

## Abstract

An open challenge of computational and experimental biology is understanding the impact of non-synonymous DNA variations on protein function and, subsequently, human health. The effects of these variants on protein stability can be measured as the difference in the free energy of unfolding (ΔΔ*G*) between the mutated structure of the protein and its wild-type form. Throughout the years, bioinformaticians have developed a wide variety of tools and approaches to predict the ΔΔ*G*. Although the performance of these tools is highly variable, overall they are less accurate in predicting ΔΔ*G* stabilizing variations rather than the destabilizing ones. Here, we analyze the possible reasons for this difference by focusing on the relationship between experimentally-measured ΔΔ*G* and seven protein properties on three widely-used datasets (S2648, VariBench, Ssym) and a recently introduced one (S669). These properties include protein structural information, different physical properties and statistical potentials. We found that two highly used input features, i.e., hydrophobicity and the Blosum62 substitution matrix, show a performance close to random choice when trying to separate stabilizing variants from either neutral or destabilizing ones. We then speculate that, since destabilizing variations are the most abundant class in the available datasets, the overall performance of the methods is higher when including features that improve the prediction for the destabilizing variants at the expense of the stabilizing ones. These findings highlight the need of designing predictive methods able to exploit also input features highly correlated with the stabilizing variants. New tools should also be tested on a not-artificially balanced dataset, reporting the performance on all the three classes (i.e., stabilizing, neutral and destabilizing variants) and not only the overall results.

## 1 Introduction

Non-synonymous DNA variations can affect the stability of the protein structure, jeopardising its function with potential pathogenic outcomes (Casadio et al., 2011; Yue et al., 2005; Hartl, 2017; Martelli et al., 2016; Cheng et al., 2008; Compiani and Capriotti, 2013; Birolo et al., 2021). For this reason, the impact of these variations on the protein structure and its stability is a widely studied problem, even though its *in silico* prediction is still challenging for bioinformaticians.

The effects of non-synonymous variants on the protein stability are usually expressed as the difference in the Gibbs free energy of unfolding (ΔΔ*G*), measured in kcal/mol and defined as the difference between the unfolding free energy of the mutated structure (*M*) of the protein and its wild-type form (*W*):

$$\Delta \Delta G_{WM}=\Delta G_{M}-\Delta G_{W}.$$ (1)

With this notation, destabilizing variants are associated to a negative ΔΔ*G*, while this value is positive for the stabilizing ones. For those mutations showing ΔΔ*G* values close to zero, the significant experimental uncertainties (Montanucci et al., 2019b; Benevenuta and Fariselli, 2019) make their ΔΔ*G* signs less reliable. At the same time, these ΔΔ*G* values indicate a minimal variation of folding stability of the variants. To account for this issue, we consider an intermediate class of variants named “neutral”, whose ΔΔ*G* values are in the range between -0.5 and 0.5 kcal/mol. The choice of 0.5 kcal/mol is based on the average experimental error, as reported in Capriotti et al. (2008).

Over the years, several computational tools have been developed to predict the ΔΔ*G*, combining machine learning methods, statistical potential energy, physico-chemical properties, sequence features and evolutionary information (Benevenuta et al., 2021; Pancotti et al., 2021; Montanucci et al., 2019a; Pires et al., 2014b; Worth et al., 2011; Samaga et al., 2021; Pires et al., 2014a; Rodrigues et al., 2018; Rodrigues et al., 2021; Schymkowitz et al., 2005; Li et al., 2021; Cheng et al., 2006; Kellogg et al., 2011; Capriotti et al., 2005; Li et al., 2020; Chen et al., 2020; Dehouck et al., 2011; Laimer et al., 2016; Savojardo et al., 2016; Savojardo et al., 2019). As highlighted in Sanavia et al. (2020), most of these tools still provide over-optimistic performance due to sequence similarity between the proteins used in the training and test sets. To provide a more realistic estimate of their performance, we recently compared the predictive ability of 18 popular tools on a novel manually-curated dataset in Pancotti et al. (2022). This dataset, named S669, was extracted from ThermoMutDB (Xavier et al., 2021) and it contains only variants belonging to proteins having less than 25% sequence identity with those of S2648 (Pires et al. (2016)) and VariBench (Nair and Vihinen (2013)), two datasets on which most of the state-of-the-art methods were trained. Our analysis underlined that, across all the methods, the performance is more accurate in predicting destabilizing variants rather than the stabilizing ones.

As possible solutions to this issue, methods had accounted for unbalanced training datasets by “artificially balancing” it or by exploiting the antisimmetry property, which is a relationship imposed on ΔΔ*G* values by thermodynamics. Specifically, given the wild-type *W* and the mutated *M* protein structures, the folding free energy ΔΔ*G*_*WM*_ from *W* to *M* is equal and has the opposite sign of the folding free energy ΔΔ*G*_*MW*_ from *M* to *W*, considering identical experimental conditions:

$$\Delta \Delta G_{WM}=\Delta G_{M}-\Delta G_{W}=-\left(\Delta G_{W}-\Delta G_{M}\right)=-\Delta \Delta G_{MW}.$$ (2)

Using this property, any unbalanced training dataset can be “artificially balanced” by introducing the reverse variants, which are substitutions created from the experimentally-measured variants, henceforth named “direct”. Considering the mutation from *W* to *M* as the “direct” variant, its “reverse” is simply defined as:

$$M\to W\text{, with }\Delta \Delta G_{MW}=-\Delta \Delta G_{WM}.$$ (3)

By artificially balancing the training dataset or by enforcing the antisymmetric property in the model itself, a method should theoretically be able to predict stabilizing variations with the same accuracy as the destabilizing ones. This statement has been already verified on S669 when considering both direct and reverse variants (Pancotti et al., 2022). Here we showed that, when considering only the direct variants, the performance is highly unbalanced with the direct stabilizing variants, which were predicted much worse than the direct destabilizing ones. Investigating the reasons for this discrepancy is the main objective of the present work.

For most methods, this imbalance could be due to any reason concerning the tool architecture or to the training phase. It is difficult, in these cases, to isolate where the problem lies. On the other hand, for an untrained method such as DDGun3D Montanucci et al. (2019a), whose prediction is a linear combination of its features, this issue can only arise from the features themselves.

For these reasons, we decided to study the impact on the predictions of the following properties of residue substitutions: the difference in hydrophobicity and volume, the logarithm of the conservation ratio, the Blosum62 evolutionary score, the relative solvent accessibility and the Skolnick and Bastolla-Vendruscolo potentials. These are the most common features considered by the state-of-the-art methods and they include the DDGun3D input features.

In this study, we showed that some of these commonly-used features are only useful to predict destabilizing variants and unhelpful for the stabilizing ones. Our findings highlight an intrinsic difference between these two classes, suggesting the importance of using different properties for the stabilizing variants in order to develop methods with more consistent performance between variant classes.

## 2 Materials and methods

### 2.1 Structural information, physical properties and statistical potentials

We considered seven different features that include the DDGun3D inputs and two more properties (conservation and volume of the amino acids involved). All these features are not specific only to DDGun3D, but they are commonly employed by ΔΔ*G* predictors. We analyzed:

1) two physical properties:

• **Difference in hydrophobicity**: the difference in hydrophobic regions between wild-type and mutant residues according to the Kyte-Doolittle scale (Kyte and Doolittle, 1982);
• **Volume difference**: the volume difference between the wild-type and the mutated residue measured in ${\overset{{}^{\circ}}{A}}^{3}$ (Zamyatnin, 1972).

2) three features based on conservation and structural information:

• **Logarithm of the conservation ratio**, defined as $\log\left(\frac{{CONS}_{W}+\epsilon }{{CONS}_{M}+\epsilon }\right)$ , with *ϵ* = 0.01 to avoid invalid results when any of the conservation frequencies were equal to zero. If the mutation changes an amino acid into a less conserved one, the logarithm value is <0 ;
• **Blosum evolutionary score**: the difference between the wild-type and mutant residues in the Blosum62 substitution matrix, *B*(*W*, *M*) (Henikoff and Henikoff, 1992);
• **Relative Solvent Accessibility**: a measure of the extent of burial or exposure of the residue in the 3D protein structure, ranging from 0 for completely buried to one for completely exposed. It was computed through the DSSP program (Kabsch and Sander, 1983; Touw et al., 2015).

3) two features based on the statistical potentials:

• **Skolnick potential**: the difference in the interaction energy (measured through the Skolnick et al. (1997) statistical potential) between the wild-type and substituted residues with their sequence neighbours within a 2-residue window

$$\overset{i=2}{\underset{i=-2}{\sum }}\left(sk\left(W,a_{i}\right)-sk\left(M,a_{i}\right)\right);$$

• **Bastolla-Vendruscolo potential**: the difference in the interaction energy, measured as the Bastolla statistical potential (Bastolla et al., 2001) between the wild-type and mutant residues with its structural neighbours,

$$\underset{i\in I}{\sum }\left(bv\left(W,a_{i}\right)-bv\left(M,a_{i}\right)\right);$$

where *I* is the set of amino acid residues in the structural neighbourhood of radius 5 $\overset{{}^{\circ}}{A}$ around the substituted position.

### 2.2 Datasets

We divided the analysis in two parts. Firstly, we studied the abilities to predict the stabilizing variations of 18 protein stability predictors on:

• **S669** (Pancotti et al., 2022) a recent manually-curated dataset extracted from ThermoMutDB (Xavier et al., 2021) whose variants belong to proteins having less than 25% sequence identity with those of S2648 and VariBench.

Secondly, we studied the correlation and the predictive ability of each different feature on a dataset that we named **S4428**, given by the combination of S669 and:

• **Ssym** (Pucci et al., 2018) which provides 684 balanced (i.e., half direct and half reverse) variations;
• **S2648** (Dehouck et al., 2011) and **VariBench** (Nair and Vihinen, 2013), two of the most used datasets, both extracted from Protherm (Kumar et al., 2006) database. They contain respectively 2,648 and 1,420 manually curated variants with experimentally measured ΔΔ*G* values.

**FIGURE 1:**
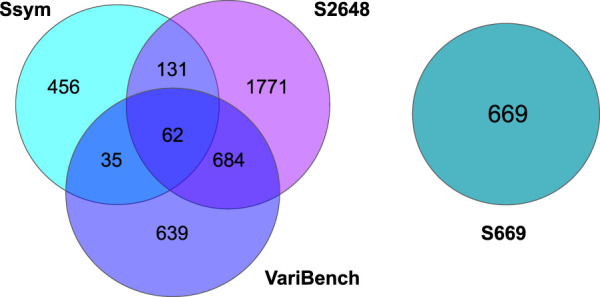
Venn diagrams showing the number of shared variants among the Ssym, VariBench, S2648 and S669 datasets.

**FIGURE 2:**
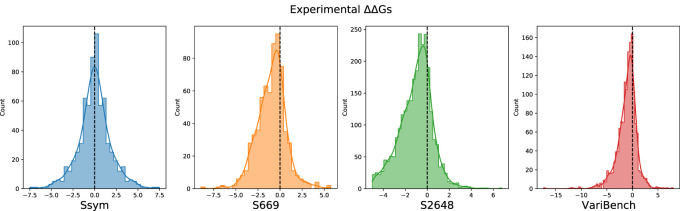
Distribution of the experimental ΔΔ*G* values in the Ssym, S669, S2648 and VariBench datasets.

After merging all the datasets, we excluded 19 variants from the analysis due to errors in their 3D neighbors. The composition of all the datasets, their intersection and the distribution of their ΔΔ*G*s are reported in Table 1, Figures 1, 2, respectively.

**TABLE 1:** Datasets composition. The variants are grouped according to their ΔΔ*G* values into three classes: destabilizing (ΔΔ*G* ≤−0.5 kcal/mol), neutral (|ΔΔ*G*| < 0.5 kcal/mol) and stabilizing (ΔΔ*G* ≥ 0.5 kcal/mol). The corresponding percentages are reported into brackets.

	Destabilizing	Neutral	Stabilizing
S2648	1,597 (60%)	755 (29%)	295 (11%)
S669	387 (58%)	195 (29%)	85 (13%)
Ssym	225 (33%)	234 (34%)	225 (33%)
VariBench	800 (56%)	426 (30%)	194 (14%)
S4428	2,461 (55%)	1,311 (30%)	656 (15%)

### 2.3 The unbalanced predictions of the state-of-the arts methods on never-before-seen variants

As shown in Figure 1, Ssym, S2648 and VariBench, which are the datasets most commonly used to train and test predictive methods, share a large number of variants. On the other hand, S669 has no variants in common with the other three datasets and its variants lie in proteins with less than 25% of sequence identity with the proteins in the other three datasets.

Since these characteristics are required for a proper test set, we will only assess ΔΔ*G* prediction performance on the S669 dataset and not on the much larger S4428. The methods’ performance in the different variant classes were evaluated by Pearson’s correlation coefficient (*ρ*) and root mean square error (RMSE) between the experimental and the predicted ΔΔ*G* (Table 2), defined as:

$$\rho =\frac{Cov\left(\Delta \Delta G^{exp},\Delta \Delta G^{\mathrm{pred}}\right)}{\sigma_{\Delta \Delta G^{exp}}\;\sigma_{\Delta \Delta G^{\mathrm{pred}}}}$$ (4)

$$RMSE=\sqrt{\frac{\sum_{i=1}^{N}{\left(\Delta \Delta G_{i}^{exp}-\Delta \Delta G_{i}^{\mathrm{pred}}\right)}^{2}}{N}}.$$ (5)

where *Cov* is the covariance, *σ* is the standard deviation and *N* is the number of variants.

**TABLE 2:** Pearson’s correlations and root mean square error (RMSE) between the experimental and estimated ΔΔ*G*s. The ΔΔ*G*s are predicted by 18 commonly used protein stability prediction tools on the direct variants of the S669 dataset. The correlations and RMSE are calculated on each class separately (“Destabilizing”-“Neutral”-“Stabilizing”), on the whole dataset (“Total”) and only on the destabilizing and stabilizing variants, excluding the neutral (“Non-neutral”).

	Pearson/RMSE				
Dataset	Total	Destabilizing	Neutral	Stabilizing	Non-neutral
MAESTRO	0.50/1.44	0.42/1.46	−0.01/0.84	0.28/2.26	0.48/1.63
ACDC-NN	0.46/1.49	0.34/1.60	0.09/0.69	0.09/2.14	0.44/1.71
INPS3D	0.43/1.50	0.35/1.40	0.03/1.00	0.02/2.55	0.42/1.67
DDGun3D	0.43/1.60	0.32/1.69	0.13/0.94	0.13/2.22	0.41/1.80
INPS-Seq	0.43/1.52	0.26/1.56	0.10/0.92	0.13/2.25	0.42/1.70
ACDC-NN-Seq	0.42/1.53	0.28/1.64	0.08/0.76	0.07/2.18	0.40/1.75
PremPS	0.41/1.51	0.43/1.48	−0.02/0.84	−0.08/2.53	0.04/1.72
PopMusic	0.41/1.51	0.37/1.40	0.11/0.96	0.09/2.67	0.39/1.69
DUET	0.41/1.52	0.34/1.48	0.02/0.89	0.10/2.54	0.38/1.72
Dynamut	0.41/1.60	0.32/1.81	0.16/0.66	0.29/2.00	0.40/1.85
SDM	0.41/1.67	0.33/1.81	0.16/1.01	0.09/2.14	0.40/1.88
DDGun	0.40/1.75	0.25/1.75	0.12/1.29	0.11/2.46	0.39/1.90
SAAFEC-Seq	0.36/1.54	0.31/1.48	0.03/0.87	0.07/2.60	0.34/1.74
mCSM	0.36/1.54	0.30/1.42	−0.01/0.96	0.06/2.73	0.33/1.73
I-Mutant3.0	0.36/1.54	0.31/1.48	0.03/0.87	0.07/2.60	0.34/1.74
I-Mutant3.0-Seq	0.34/1.56	0.23/1.53	0.05/0.92	0.21/2.53	0.33/1.75
MuPro	0.25/1.61	0.19/1.45	0.08/1.08	−0.01/2.84	0.24/1.78
FoldX	0.21/2.32	0.20/2.25	0.01/2.28	0.17/2.66	0.24/2.33

### 2.4 Assessing the impact of the input features in the prediction of destabilizing, neutral and stabilizing variants

In order to assess the relevance of the seven features listed in Section 2.1, we computed the Pearson’s correlation coefficient (*ρ*) between each feature and the experimental ΔΔ*G*s in S4428 (Table 3). To remove the bias in the *ρ* values on the whole dataset caused by the under-representation of the stabilizing and neutral variants with respect to the destabilizing ones (Table 1), we randomly under-sampled the available data to balance the classes. Specifically, we generated 100 random balanced subsets of 984 variants extracted from the original dataset. For each class we selected 328 elements, half the size of the smallest class of variants on S4428. For each feature, the average Pearson’s correlation coefficient and its standard deviation are reported in Table 3 (column “Total balanced”).

**TABLE 3:** Pearson correlation coefficient (*ρ*) between the experimental ΔΔ*G*s and the seven considered features in the S4428 dataset. On the column “Total”, we have the correlation in the whole unbalanced dataset, while on the column “Total Balanced” we reported the average Pearson’s correlation coefficient and its standard deviation calculated in 100 random subsets with the same number of variants for each class (Stabilizing-Neutral-Destabilizing). The last three columns report the correlation on each class separately, considering all the possible variants.

	Pearson’s correlation coefficient				
	Total	Total balanced	Destabilizing	Neutral	Stabilizing
Accessibility	0.22	0.08 ± 0.02	0.28	0.02	−0.21
Bastolla-Vendruscolo potential	0.45	0.45 ± 0.03	0.36	0.13	0.18
Blosum evolutionary score	0.12	0.02 ± 0.02	0.26	−0.02	−0.18
Difference in hydrophobicity	−0.21	−0.18 ± 0.03	−0.19	−0.04	−0.04
Volume difference	−0.32	−0.34 ± 0.03	−0.22	−0.12	−0.15
Logarithm of the conservation ratio	−0.37	−0.37 ± 0.02	−0.27	−0.11	−0.21
Skolnick potential	0.36	0.35 ± 0.03	0.29	0.07	0.13

We also evaluated the impact of the seven features in terms of their discriminative power using the Receiver Operating Characteristic (ROC) curve and its Area-Under-the-Curve (AUC-ROC) metric. We removed the size effect by under-sampling the variants 100 times in order to have 100 subsets of 656 variants for each pair of classes. On these under-sampled datasets we used each feature to calculate three different ROC curves separating three pairs of classes: Destabilizing-Neutral, Destabilizing-Stabilizing, Neutral-Stabilizing. Here, the assumption is that the higher the AUC scores, the better the separation of the two classes by the variable and, therefore, the more informative the variable is. The results of this analysis are shown in Table 4 and in the Supplementary Materials. The ROC curves and AUC scores of the logarithm of the conservation ratio, the volume difference and the difference in hydrophobicity were calculated using the values with opposite signs to help the interpretation. In Table 4, we also reported the *p*-values of the two-sided Mann-Whitney Wilcoxon test computed between the distributions of the scores for each pair of classes.

**TABLE 4:** Ability of each feature to separate the different classes and differences in distributions. We extracted one hundred subsets of size 328 from each class and used the variable score to calculate three different ROC curves: Destabilizing-Neutral, Destabilizing-Stabilizing, Neutral-Stabilizing. The assumption, here, is that the higher the AUC-ROC, the better the variable can separate between the two classes and the more informative it is. For each pair of classes we also computed the Mann-Whitney-Wilcoxon test two-sided to establish the statistical significance of the differences in the distributions.

AUC-ROC scores and *p*-values						
	Destabilizing-neutral		Destabilizing-stabilizing		Neutral-stabilizing	
	*AUC* ± *std*	*p-values*	*AUC* ± *std*	*p-values*	*AUC* ± *std*	*p-values*
Accessibility	0.69 ± 0.02	1.98e-79	0.55 ± 0.02	1.43e-04	0.36 ± 0.01	1.18e-23
Bastolla-Vendruscolo potential	0.66 ± 0.02	1.40e-58	0.75 ± 0.01	8.50e-84	0.63 ± 0.02	1.26e-22
Blosum evolutionary score	0.58 ± 0.02	5.61e-17	0.50 ± 0.02	7.08e-01	0.42 ± 0.02	1.21e-09
Difference in hydrophobicity	0.59 ± 0.02	9.41e-22	0.60 ± 0.02	2.28e-16	0.51 ± 0.02	4.22e-01
volume difference	0.60 ± 0.02	6.90e-24	0.72 ± 0.02	1.24e-63	0.64 ± 0.02	1.02e-22
Logarithm of the ratio of the conservation	0.66 ± 0.02	1.00e-58	0.75 ± 0.01	1.32e-84	0.63 ± 0.02	1.29e-20
Skolnick potential	0.64 ± 0.02	2.56e-41	0.71 ± 0.02	4.27e-63	0.60 ± 0.02	4.97e-14

### 2.5 Training a predictor with the reduced set of features

To evaluate the combined effect of the Blosum evolutionary score, difference in hydrophobicity and accessibility on the predictions of the different classes, we trained a Random Forest regressor on 100 random balanced subsets of S4428 (excluding S669) and then tested its performance on S669 (Table 5). We used three different sets of features: one with all the seven variables (“full”), a reduced set with all the variables except for Blosum evolutionary score and difference in hydrophobicity (“reduced”) and one where we also excluded the accessibility (“red-no-acc”). Therefore, we tested to which extent the removal of the evolutionary-based component, the hydrophobicity and the accessibility affects DDGun3D predictions. The original DDGun3D score was computed as:

$${\text{DDGun}3\text{D}}_{\text{full}}=\left(\left.0.20\cdot S_{Bl}+0.29\cdot S_{Sk}+0.18\cdot S_{Hp}+0.33\cdot S_{BV}\right]\right)\cdot \left(1.1-acc\right),$$ (6)

where *S*_*Bl*_, *S*_*Sk*_, *S*_*BV*_, *S*_*Hp*_, *acc* are, respectively, the components related to the Blosum evolutionary score, the Skolnick and the Bastolla-Vendruscolo potentials, the difference in hydrophobicity and the accessibility, with the same coefficient and definition as in the original paper Montanucci et al. (2019a). We defined two “reduced” scores by dropping the corresponding components:

$$\begin{array}{cc} {\text{DDGun}3\text{D}}_{\text{reduced}} & =\left(\left.0.29\cdot S_{Sk}+0.33\cdot S_{BV}\right]\right)\cdot \left(1.1-acc\right),\\ {\text{DDGun}3\text{D}}_{\text{red}-\text{no}-\text{acc}} & =\left(\left.0.29\cdot S_{Sk}+0.33\cdot S_{BV}\right]\right). \end{array}$$ (7)

**TABLE 5:** Predictions with different sets of features. We tested to which extent the removal of the Blosum component, the hydrophobicity and the accessibility affects the performance of a Random Forest regressor and DDGun3D on S669. We used three different sets of features: one with all the seven variables, one with all the variables except for Blosum evolutionary score and the difference in hydrophobicity (“reduced”) and one where we also excluded the accessibility (“red-no-acc”). Results are reported in terms of Pearson correlation coefficient and RMSE.

	Pearson/RMSE		
	Total	Destabilizing	Stabilizing
DDGun3D_full_	0.43/1.6	0.32/1.69	0.13/2.22
DDGun3D_reduced_	0.37/1.69	0.29/1.96	0.17/1.96
DDGun3D_red-no-acc_	0.34/1.75	0.26/2.02	0.15/2.0
Random Forest_full_	0.42/1.56	0.28/1.70	0.15/2.09
Random Forest_reduced_	0.40/1.58	0.27/1.72	0.16/2.15
Random Forest_red-no-acc_	0.4/1.58	0.26/1.71	0.19/2.13

## 3 Results

We divided the results into four sections: the first shows the methods’ performance on the different variant classes on S669, a blind testing set, the second explores the correlation of the seven features with the experimental ΔΔ*G* values, the third section investigates their discriminative power and the fourth section analyze their combined impact on the predictions.

### 3.1 The unbalanced predictions among stabilizing, neutral and destabilizing variants

In a previous study (Pancotti et al., 2022) we assessed the performance of 18 ΔΔ*G* prediction methods on the S669 dataset, a test dataset with no intersection with the methods’ training sets and whose variants are in proteins with less than 25% of sequence identity with those in the training sets. We used both direct and reverse variants (see Introduction) to show that, when dealing with never-before-seen variants, those methods that do not respect the antisymmetry property have a worse performance on stabilizing variants, while the antisymmetric ones performed consistently well on both classes.

In this study, we focused uniquely on direct variants and we showed that the difference in the non-antisymmetric methods’ performance between destabilizing and stabilizing variants is even bigger when we do not consider the reverse variants. In addition, even antisymmetric methods showed a highly uneven performance between the classes (Table 2). The correlations on the whole dataset and on the non-neutral variants (destabilizing and stabilizing) were relatively good, with most methods having *ρ* ≥ 0.4, showing that the predictions captured the general trend of the experimental ΔΔ*G*s. Most methods also predicted relatively well the destabilizing variants, with a *ρ* ≥ 0.3. None of the methods, however, was able to predict the stabilizing variants with a good correlation. The highest Pearson’s correlation on the stabilizing variants was ≤0.3 and the lowest RMSE was ≥2 .

This result confirms the difficulty of current methods in adequately recognizing the direct-stabilizing variants.

### 3.2 Relevance of the seven features: Correlation on S4428

We assessed the relevance of the seven features of interest by computing their Pearson’s correlation coefficient (*ρ*) with the experimental ΔΔ*G*s considering the S4428 dataset (Table 3). When considering the whole dataset, all the features showed absolute correlations ranging from *ρ* = 0.12, observed with the Blosum evolutionary score, to *ρ* = 0.45, reached by the Bastolla-Vendruscolo potential (Table 3, column “Total”).

However, when the different classes were balanced by randomly sampling 100 balanced subsets of ∼1,000 variants (column = “Total balanced”), the correlation dropped significantly for the Blosum evolutionary score and for the accessibility, since these two features are correlated with the destabilizing variants (*ρ* = 0.26 and *ρ* = 0.28, respectively) but anti-correlated with the stabilizing ones (*ρ* = −0.18 and *ρ* = −0.21, respectively). The reason for the anti-correlation of the Blosum score with stabilizing variants and the correlation with the destabilizing ones is the symmetry of the score, since *B*(*W*, *M*) = *B* (*M*, *W*). On the other hand, for the accessibility this happens because, in general, the more one amino acid is buried, the greater impact its mutation has on the stability of the protein (either stabilizing or destabilizing). Therefore, this feature should be used to modulate the impact of the mutation, not to predict the sign.

**FIGURE 3:**
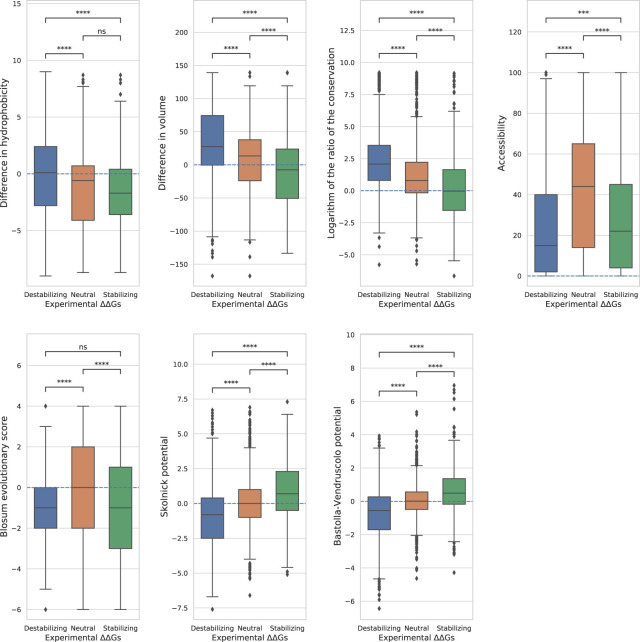
**Distributions of the features**. Boxplots showing the distributions of the features on the three classes. The variations are considered neutral if ΔΔ*G* ∈[−0.5, 0.5], stabilizing if ΔΔ*G* <−0.5 and destabilizing if ΔΔ*G* > 0.5. For each pair of classes we computed the Mann-Whitney-Wilcoxon test two-sided to establish the difference in the distributions. The *p*-values are reported here in a compact way: “ns” - *p* > 0.05, * - 0.01 < *p* ≤ 0.05, ** - 1.0*e*−03 < *p* ≤ 0.01, *** - 1.0*e*−04 < *p* ≤ 1.0*e*−03, **** - *p* ≤ 1.0*e*−04, the actual values of the *p*-values are in Tab.4.

The anti-correlation observed between the experimental ΔΔ*G* and the logarithm of the conservation ratio is coherent with the assumption that the substitution of an amino acid with a less conserved one $\left(\log\left(\frac{{CONS}_{W}}{{CONS}_{M}}\right)>0\right)$ will likely have a disruptive effect, while substituting it with a more conserved one $\left(\log\left(\frac{{CONS}_{W}}{{CONS}_{M}}\right)<0\right)$ will likely have a stabilizing effect. The volume difference and the hydrophobicity between the two amino acids involved were also anti-correlated with the experimental ΔΔ*G*, meaning that a big absolute volume difference is associated with a greater effect, with a positive difference (i.e., mutated amino acid larger than the wild-type) being disruptive and a negative difference (i.e., wild-type greater than the mutated) being stabilizing. For hydrophobicity, while there seems to be a general trend (Figure 3, *ρ* = −0.21 on the whole dataset), neutral and stabilizing variants showed little correlations (*ρ* = −0.04 for both).

### 3.3 Discriminative power of the seven features: AUC-ROC on S4428

We also evaluated the discriminative power of the seven features using the AUC-ROC metric. We assumed that a specific feature is informative and useful for the prediction tools if it is able to separate the three pairs of classes (Destabilizing-Neutral, Destabilizing-Stabilizing, Neutral-Stabilizing). The higher the AUC-ROC scores for these separations, the more informative the feature is.

To remove the size effect, we under-sampled the variants 100 times in order to have 100 subsets of 656 variants for each pair of classes. In addition, due to their anti-correlation with the ΔΔ*G*s, the ROC curves of the logarithm of the conservation ratio, the volume difference and the difference in hydrophobicity were calculated using the values with opposite signs to respect the monotonic assumption that higher AUC-ROC means higher discriminative power, making them more immediately interpretable to the reader. In this way, instead of having a score of e.g.,: 0.3, the score would become 1–0.3 = 0.7. All the distributions of the features and the statistical differences between the classes computed using the two-sided Mann-Whitney Wilcoxon test are displayed in Figure 3 and in Table 4. The average AUC scores with their standard deviations are reported in Table 4, while the ROC curves are displayed in the Supplementary Materials.

The boxplots clearly show that none of the features perfectly separates all the three classes (Figure 3).

Overall, we found that the features with the highest absolute Pearson’s correlation coefficients on the balanced datasets (Table 3, “Total balanced”) were the best at separating between destabilizing and stabilizing variants, while those with a poor correlation also showed a poor discriminative power. The AUC scores “Destabilizing-Stabilizing” of the Bastolla-Vendruscolo potential, the Skolnick potential, the logarithm of the conservation ratio and the volume difference were all greater than 0.7, reflecting their high correlations on the balanced dataset. On the other hand, the accessibility and the difference in hydrophobicity, which are characterized by a low correlation on the balanced datasets (0.08 and −0.18, respectively), also showed low AUCs when separating destabilizing variants from stabilizing (0.55 and 0.6, respectively). The Blosum evolutionary score, which was uncorrelated on the balanced dataset, showed also random behaviour (AUC = 0.5) in separating the destabilizing from the stabilizing variants and the two distributions were not significantly different (*p*-value of Mann-Whitney test = 0.71). A non-significant *p*-value (*p* = 0.4) was also observed for the difference in hydrophobicity between stabilizing and neutral variants. This feature, while being able to slightly separate the destabilizing variants from the other two classes (*AUC* = 0.59 and *AUC* = 0.6), was not able to separate stabilizing from neutral variants (AUC = 0.51).

The accessibility separated fairly well the neutral variants from the other two classes, but not the destabilizing from the stabilizing (Figure 3), suggesting that it should only be used as a modulator of the ΔΔ*G* absolute value. For the other best performing features, the two potentials and the conservation showed sightly higher similarity between neutral and stabilizing variants than between neutral and destabilizing, while the volume difference showed an opposite trend (Figure 3).

### 3.4 Improving the predictions on the stabilizing variants

We evaluated the effects of including or excluding the Blosum evolutionary score, the difference in hydrophobicity and the accessibility in a Random Forest predictor and in DDGun3D. The aim of this analysis was not to outperform existing methods, but to analyze how the combination of these variable affects the predictions on the different classes.

Table 5 shows the results obtained by these two predictors when using three possible sets of features: “all variables”, “reduced”, “red-no-acc”. “Reduced” includes all variables except for Blosum evolutionary score and difference in hydrophobicity, while “red-no-acc” also excludes the accessibility.

The results show that, excluding the Blosum evolutionary score and difference in hydrophobicity, the correlation decreases on the destabilizing class for both methods: *ρ* = 0.32 for DDGun3D and *ρ* = 0.28 for Random Forest with “all variables”, compared to *ρ* = 0.29 and 0.27 with the “reduced” set. The same behaviour in the two predictors was observed when the “red-no-acc” set was considered. Given the high unbalance in S669 towards destabilizing mutations, with 387 variants being destabilizing and only 85 being stabilizing, the decreasing performance on the destabilizing variants affects the overall correlation on the whole dataset too.

Removing the Blosum evolutionary score and the difference in hydrophobicity, however, increases the correlation on the stabilizing class, as expected. In addition, the correlation increases when we also remove the accessibility from the Random Forest predictor’s features (*ρ* = 0.19 vs. *ρ* = 0.15), but not when DDGun3D is used (*ρ* = 0.15 vs. *ρ* = 0.13). However, it is worth noticing that, while in the Random Forest predictor the accessibility is used as any other feature, in DDGun3D it is used as modulator of the ΔΔ*G* (see Eqs 6, 7). Indeed, using the Random Forest predictor, the additional removal of the accessibility improves the performance with respect to the removal of only the Blosum evolutionary score and the difference in hydrophobicity (*ρ* = 0.19 vs. *ρ* = 0.16), while for DDGun3D this additional removal negatively affects the correlation (*ρ* = 0.15 vs. *ρ* = 0.17).

## 4 Discussion and conclusion

Our study is based on the observation that none of the tools available to predict the ΔΔ*G* is very accurate on the stabilizing variants and, in general, the predictions are skewed towards neutral and destabilizing variations. The existing datasets for ΔΔ*G* prediction share a large number of variants and are strongly unbalanced towards the destabilizing variants (Table 1; Figure 2). For this reason, choosing features that favour only the most abundant class can lead to a good overall performance at the cost of penalizing the prediction of the less abundant class.

To better understand the weakness in correctly predicting the stabilizing variants, we evaluated seven properties commonly used by the computational ΔΔ*G* predictors. We considered two features based on physical properties (hydrophobicity and volume), three structural information and conservation-based features (Blosum evolutionary score, conservation and relative solvent accessibility) and two statistical potentials-based features (Skolnick potential and Bastolla-Vendruscolo potential). For each of them, we analyzed the ability to predict the experimental ΔΔ*G* by computing the Pearson’s correlation coefficient (*ρ*) between them and the experimental ΔΔ*G*s of three of the most used dataset (S2648, VariBench and Ssym) and a recently-released one (S669), all combined in the S4428 dataset. We also computed the AUC-ROCs for each variant to judge how these features separate the different classes of variations. The results showed that the volume difference, the logarithm of the conservation ratio, and the statistical potentials are better than random in each possible separation, i.e., destabilizing vs neutral, destabilizing vs stabilizing and neutral vs stabilizing variants. On the other hand, the difference in hydrophobicity, the Blosum evolutionary score and the accessibility likely showed random results in at least one of the separations. Although hydrophobicity difference has an anti-correlation trend in the balanced dataset (*ρ* = −0.18), it cannot separate the neutral variants from the stabilizing ones (AUC-ROC = 0.51 ± 0.02), and it has marginal ability to separate destabilizing from neutrals (AUC-ROC = 0.59 ± 0.02). Thus, hydrophobicity difference alone can contribute to entangling stabilizing and neutral variants at prediction time. In turn, this may lead to enforce the role of the destabilizing variants during the method training.

Due to the symmetry of the Blosum evolutionary score, it is not surprising that the AUC-ROC in separating destabilizing and stabilizing variants was 0.5 ± 0.02. However, the score also failed to separate the other two classes from the neutral ones (AUC-ROCs = 0.58/0.42 ± 0.02), which could lead to predictions skewed towards the neutral class.

The accessibility is actually fairly good at separating the neutral from the other two classes (AUC-ROCs = 0.69/0.36 ± 0.02), but not the destabilizing from the stabilizing (AUC-ROC = 0.55 ± 0.02). Accessibility is then a good marker of the general impact of a variation (being them stabilizing or destabilizing) since its absolute value of *ρ* on the stabilizing variants (|*ρ*| = 0.21) is one of the highest among the considered features.

Among the seven features, the logarithm of the conservation ratio best described the stabilizing variants (*ρ* = −0.21), consolidating the known important role of the conservation in the impact of the genetic variants. Moreover, the logarithm of the conservation ratio and the volume difference showed the smallest drop in correlation between destabilizing and stabilizing variants (Tab.3). The other two best performing features, the Bastolla-Vendruscolo potential and the Skolnick potential, showed a bigger drop in correlation, even though the Bastolla-Vendruscolo potential by itself reached a correlation of *ρ* = 0.45 on the full dataset, which is not that far off from the results that many predictors get using way more features and information.

We tested the impact of removing Blosum62, hydrophobicity and accessibility (the three worst features at separating the stabilizing variants from the rest) on prediction by examining two models: DDGun3D, a mostly linear ΔΔ*G* predictor with limited interaction between features and a Random Forest, a fully non-linear generic method. Independently from the predictor used, dropping out Blosum62 and hydrophobicity improves the performance on stabilizing variants at the cost of a loss on destabilizing ones.

This is not surprising for a linear model where each feature has a fixed and independent effect on all variants. On the other hand, a more powerful method like Random Forest, that can model interactions and varying non-linear effects, could be able to use these features (Blosum62 and hydrophobicity) selectively only for the non-stabilizing variants for which they are informative. However, our tests suggest that, in practice, including them can be harmful for stabilizing variant prediction regardless of the model.

On the other hand, the behaviour of the accessibility is more complex since the less accessible residues tend to have a higher impact than the accessible ones. In some way, accessibility can modulate the stability effect as, without it, the performance on the stabilizing variants improves in the Random Forest model but worsens in DDGun3D when it is not used as a shrinking effect (Table 5). This result also agrees with previous studies that led to the tool PopMusic2 (Dehouck et al., 2011).

Our results suggest not incorporating the substitution matrix score (e.g., Blosum62) and the difference in hydrophobicity in future predictive computational tools, while using the difference in accessibility only to modulate the impact of the variant. Furthermore, we suggest using the features that better separate the stabilizing from the neutral variants (such as the Bastolla-Vendruscolo potential, the logarithm of the conservation ratio and the volume difference) to avoid the compression of the predictions towards the neutral values. Finally, new tools should also be tested on not-artificially balanced datasets, reporting the performance specifically for each variant class.

## Data Availability

Publicly available datasets were analyzed in this study. This data can be found here: https://academic.oup.com/bib/article/23/2/bbab555/6502552#supplementary-data.
