# Supplementary figures and images for: Challenges in predicting stabilizing variations: An exploration
Source: Front Mol Biosci. 2023 Jan 5;9:1075570. doi: 10.3389/fmolb.2022.1075570 (PMC9849384; doi:10.3389/fmolb.2022.1075570)

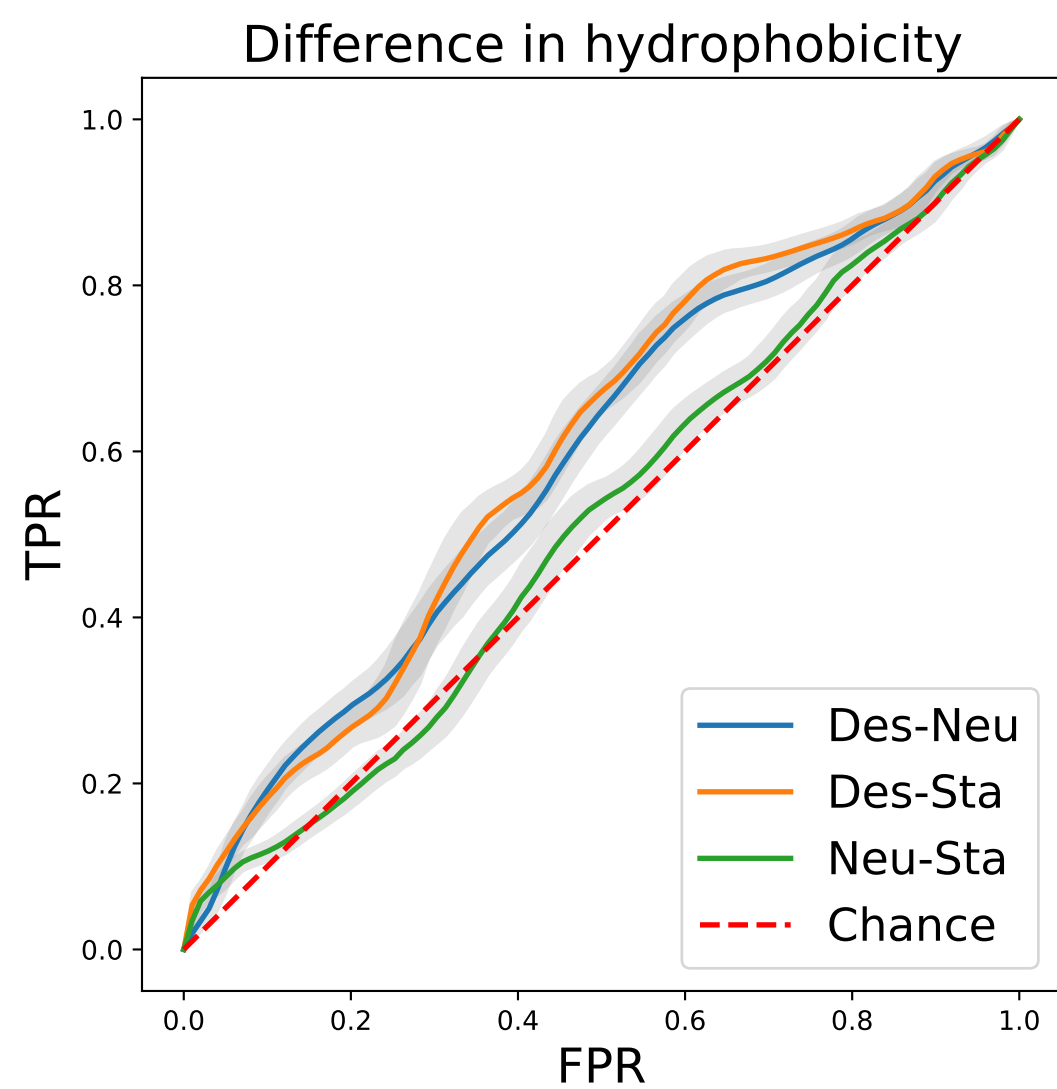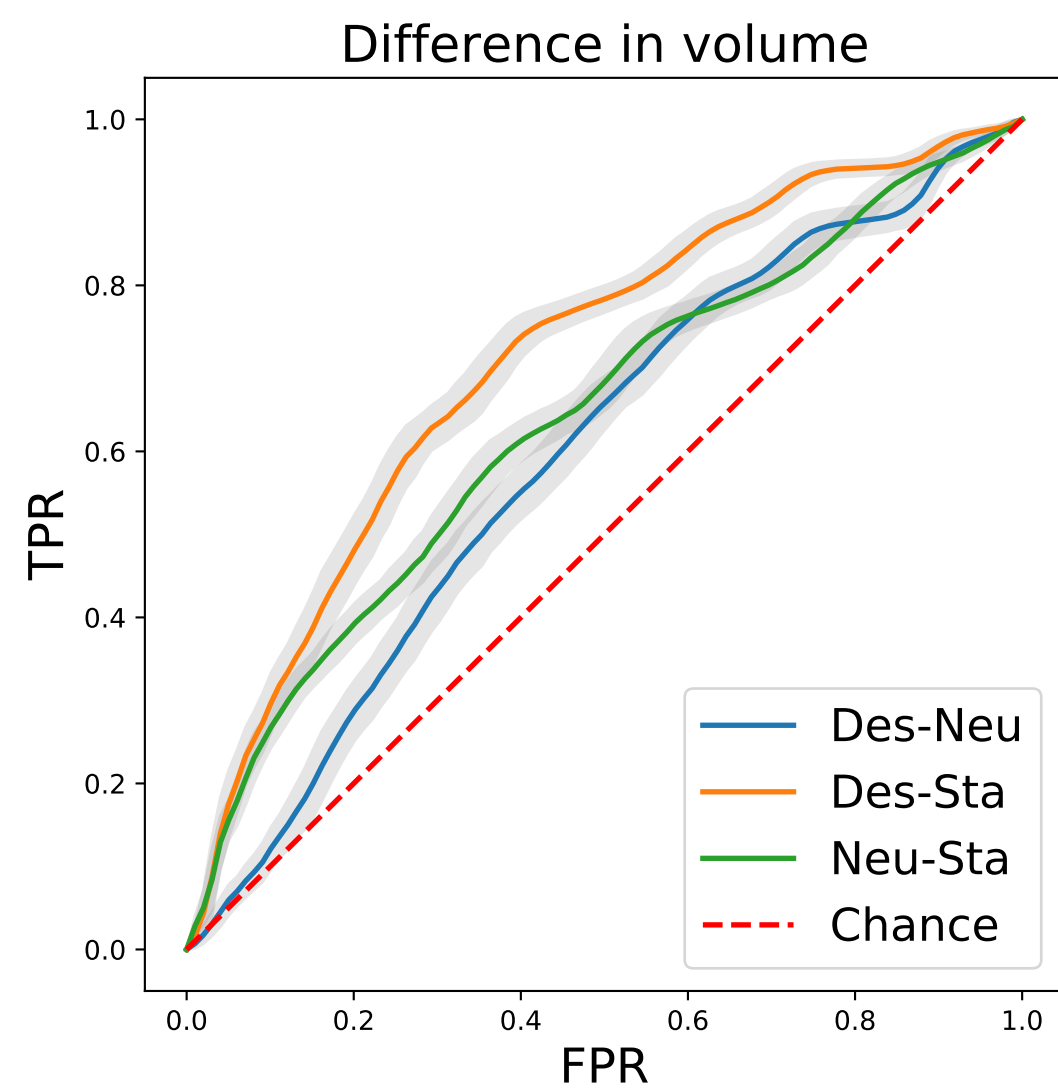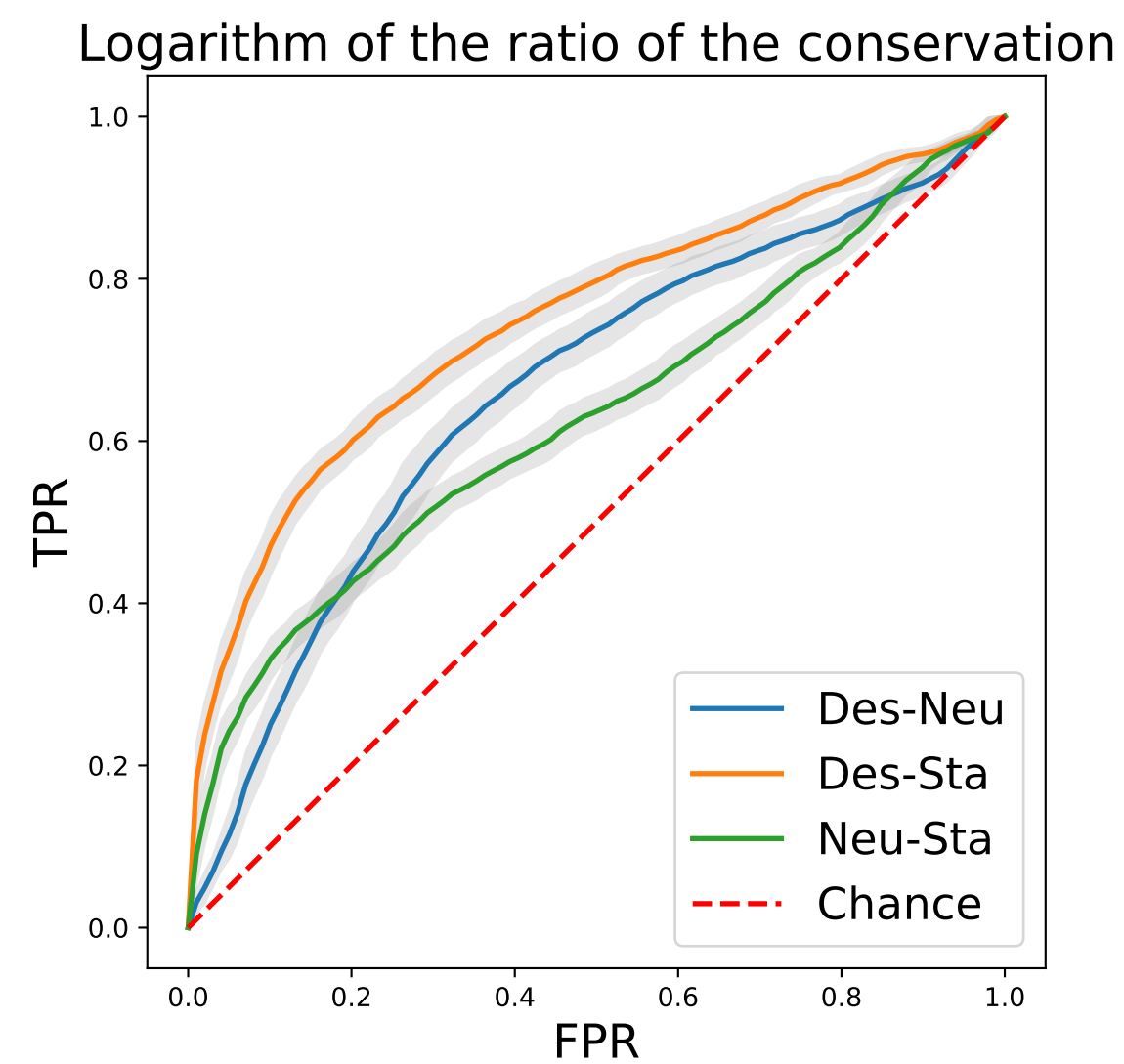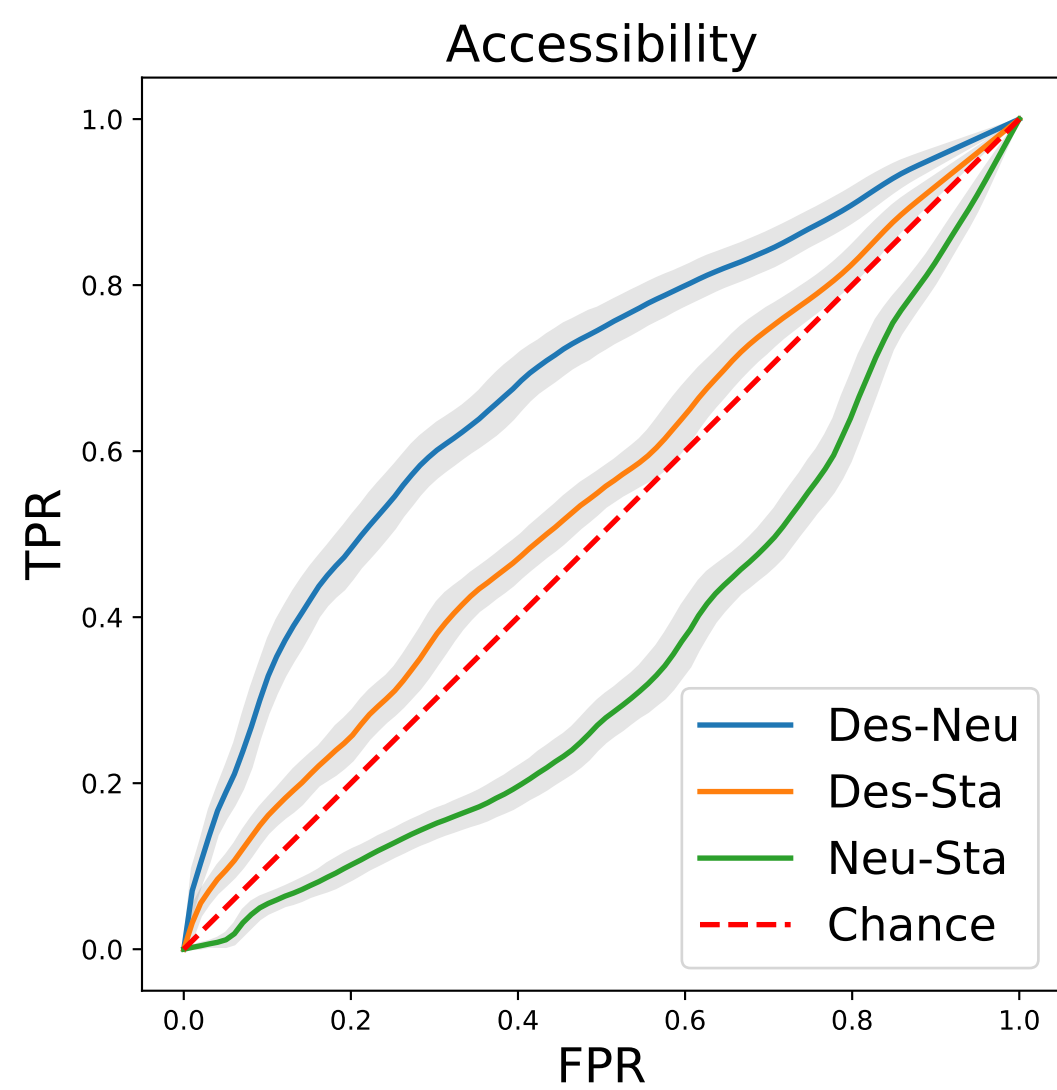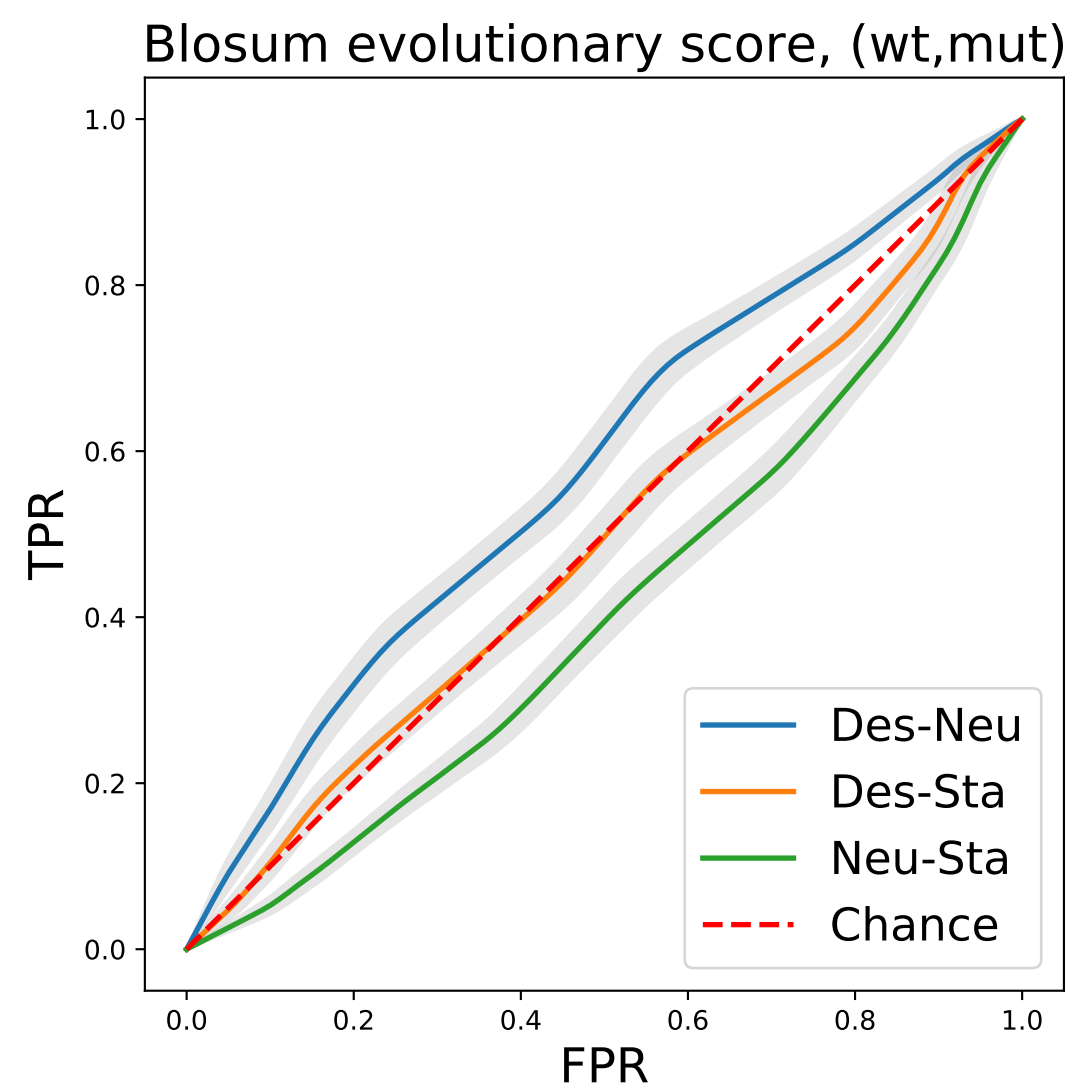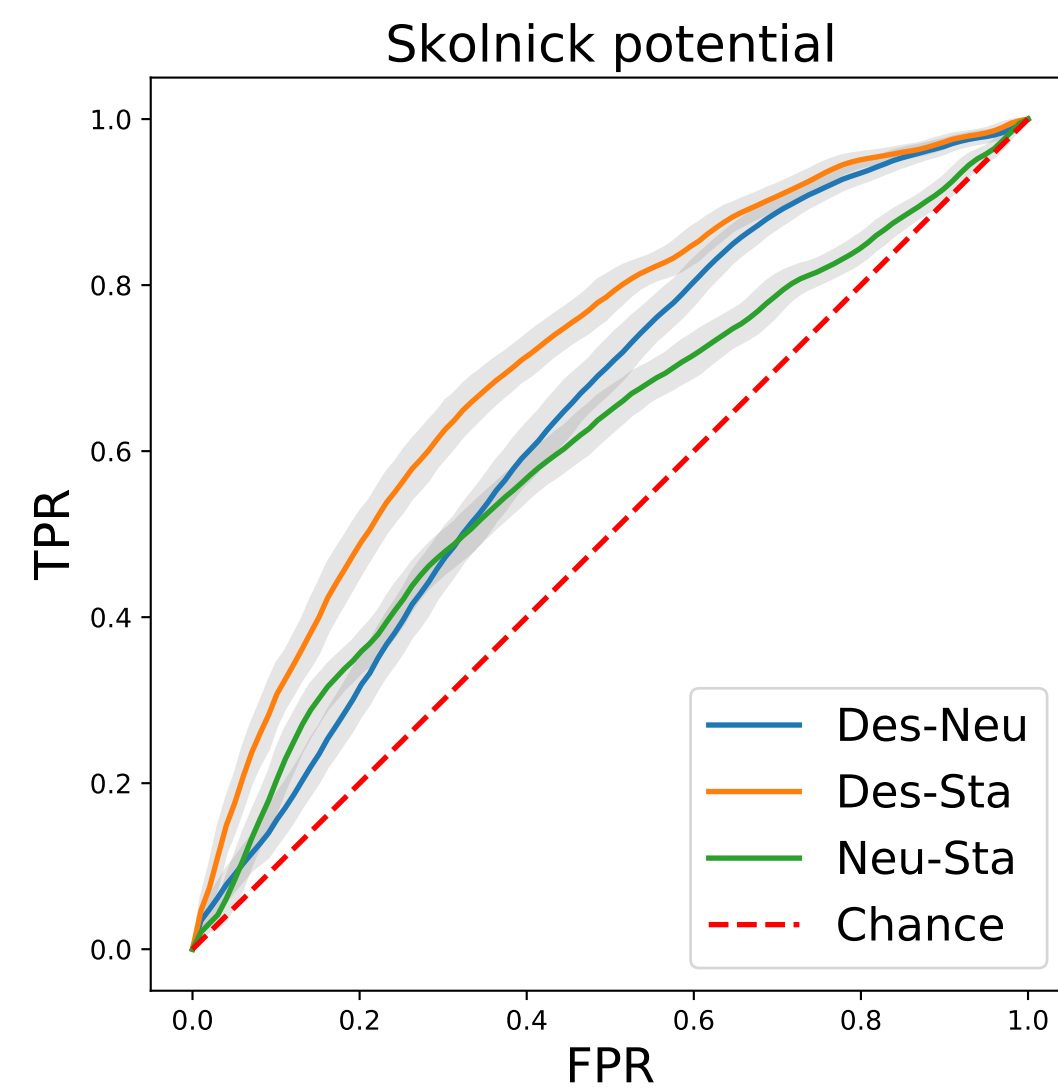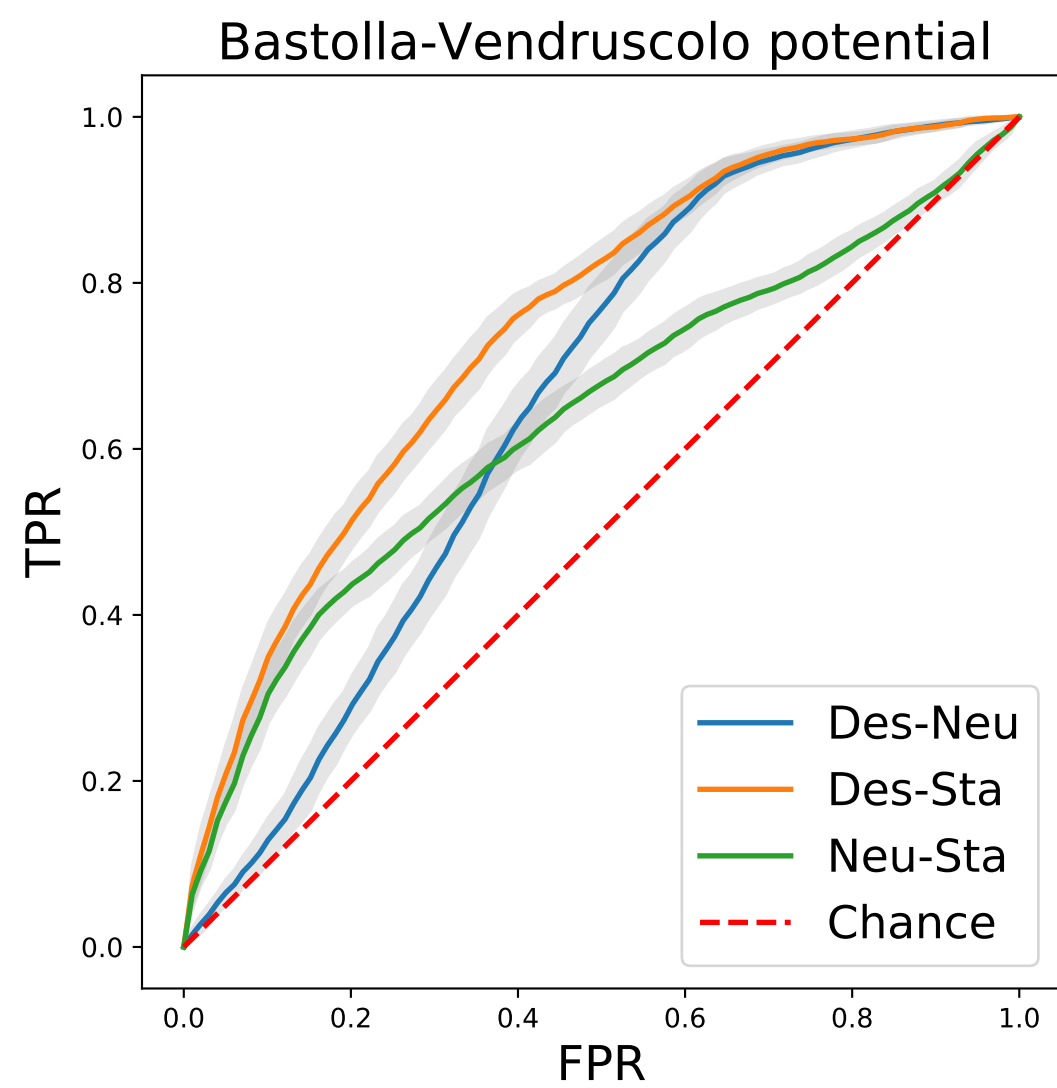

Supplement: Supplementary file 1 [file DataSheet1.PDF]
